# Barriers and facilitators to implementing Women in Surgery initiatives at UK medical schools: A qualitative study

**DOI:** 10.1016/j.sopen.2026.08.008

**Published:** 2026-08-29

**Authors:** Samruddhi Tagalpallewar, Lara Melloul, Oliwia Bogucka, Lucy Bowden, Arul Nilaa Arul Jothi, Ira Makhijani, Ranoo Rebaz, Polina Zabelina

**Affiliations:** a University College London, London, United Kingdom; b Lancaster University, Lancaster, United Kingdom; c Keele University, Keele, United Kingdom; d University of Plymouth, Plymouth, United Kingdom; e University of Nottingham, Nottingham, United Kingdom; f University of Glasgow, Glasgow, United Kingdom

**Keywords:** Women in surgery, Surgery, EDI, Equality in healthcare

## Abstract

**Background:**

Despite women comprising over half of UK medical school graduates, they represent only 17% of consultant surgeons. This imbalance limits female students' access to mentorship and career opportunities. Specialised Women in Surgery (WinS) programs at medical schools are key initiatives to address this imbalance and support aspiring female surgeons.

**Methods:**

This cross-sectional qualitative study surveyed 24 UK medical schools through semi-structured interviews with 53 representatives between October and December 2024. Data collection employed a mixed-method approach using open-ended and Likert scale questions (1–5), followed by thematic and quantitative analyses.

**Results:**

Of 24 participating UK medical schools, only 10 (41.7%) had an active or developing WinS initiative. Institutions with WinS programs reported higher engagement in gender equity and greater student awareness. The value of WinS was rated highly (4.5/5), with strong student demand (83.3%). The most significant barriers were high workload and lack of student time (mean rating: 3.33), funding constraints (mean rating: 3.01), and insufficient access to female surgeons (mean rating: 2.86). A key divergence emerged where universities without WinS programs struggled most with accessing female surgeons, while those with WinS programs identified funding as their main challenge.

**Conclusion:**

With only 41.7% of UK medical schools having established WinS initiatives, there is a critical need for coordinated national support. A framework providing shared resources and networking opportunities could transform isolated programs into an effective support system. These findings offer actionable recommendations to address inequities and empower future female surgeons.

## Introduction

Advancing gender equity remains a pressing challenge across surgical specialties, both in the UK and globally. Despite decades of progress in medical education, surgery continues to reflect deeply entrenched gender disparities, with women consistently underrepresented in leadership roles, surgical specialties, and academic appointments.

In the UK, although women constitute over 50% of medical school graduates [1], they account for only 17% of consultant surgeons [2], a stark indicator of the systemic barriers that persist. These include limited access to mentorship, a lack of visible role models, implicit bias in selection and promotion processes, and cultural norms that often dissuade women from pursuing or persisting in surgical careers [3].

To address these persistent inequities, university Women in Surgery (WinS) programs have emerged as targeted interventions to promote gender diversity and inclusion within the surgical pipeline. By providing mentorship, fostering supportive peer networks, and spotlighting female surgical role models, these programs aim to dismantle barriers and create environments where aspiring female surgeons can thrive. In doing so, WinS initiatives also contribute to broader institutional goals of promoting education on surgical training, practicing surgical skills, and student socialisation.

However, significant gaps remain in our understanding of how WinS programs are implemented across UK medical schools. There is a notable lack of data on the strategies employed by student surgical societies to promote gender equity, and no standardised framework exists to guide or unite these efforts. As such, the efficacy and sustainability of these initiatives vary widely, with many programs lacking the institutional support or structure necessary for long-term impact.

This national qualitative study addresses critical knowledge gaps by exploring the structural, cultural, and logistical factors that determine the success of Women in Surgery (WinS) programs within UK medical schools and by evaluating their impact on student engagement in surgical careers.

## Methods

### Study design

This cross-sectional qualitative study was designed to explore the barriers and facilitators to implementing Women in Surgery (WinS) programs in UK medical schools. All participants provided informed consent prior to participation, and the study adhered to the Standards for Reporting Qualitative Research (SRQR) reporting guideline [4].

Data were anonymised, and any identifiable references were removed or generalised in analysis. Member-checking was employed to enhance trustworthiness of findings, with selected participants reviewing excerpts of their data to ensure accurate representation.

### Setting

This cross-sectional study was conducted across 24 UK medical schools between October and December 2024. A total of 53 participants were recruited, including surgical society presidents, WinS leads, and other key representatives involved in gender equity initiatives. All 45 UK medical schools, recognised by the GMC at the time of survey distribution (2025), were contacted to identify eligible representatives, and a purposive sampling strategy was employed, targeting individuals with direct experience in designing, supporting, or evaluating a WinS program. The final sample ensured representation across diverse geographic regions, institutional sizes, and varying stages of surgical society infrastructure and development.

### Data collection

Interview questions were informed by previous literature and with input from student researchers experienced in WinS leadership. Semi-structured interviews (SSIs) consisted of open-ended questions exploring barriers, institutional support, mentorship, and program impact, complemented by Likert-scale ratings (1–5) on key barriers and current student engagement with gender equity. Each interview lasted approximately 30 to 45 min and was conducted via video conferencing. Follow-up questions were used to clarify responses and probe emerging themes. All interviews were audio recorded and transcribed verbatim for analysis. Informational redundancy was achieved within the sample, and no new themes emerged in the final interviews, suggesting saturation.

### Data analysis

All interviews were transcribed and imported into Microsoft Excel software for analysis. Two researchers independently reviewed and coded the transcripts, employing thematic analysis to identify key patterns and themes related to the implementation of WinS programs. An initial coding framework was developed inductively from the data, then refined through iterative discussions between coders. Discrepancies were resolved by a third researcher. To complement the thematic analysis, Likert-scale responses were analysed using descriptive statistics to quantify perceptions across the participant pool. Mann Whitney test was used to test for statistical significance.

## Results

### Overview of findings

A total of 24 UK medical schools participated in this study, with 10 (41.7%) reporting active or developing Women in Surgery (WinS) initiatives. The remaining 14 (58.3%) had no WinS programs in place.

### Impact of WinS programs

**Fig. 1:**
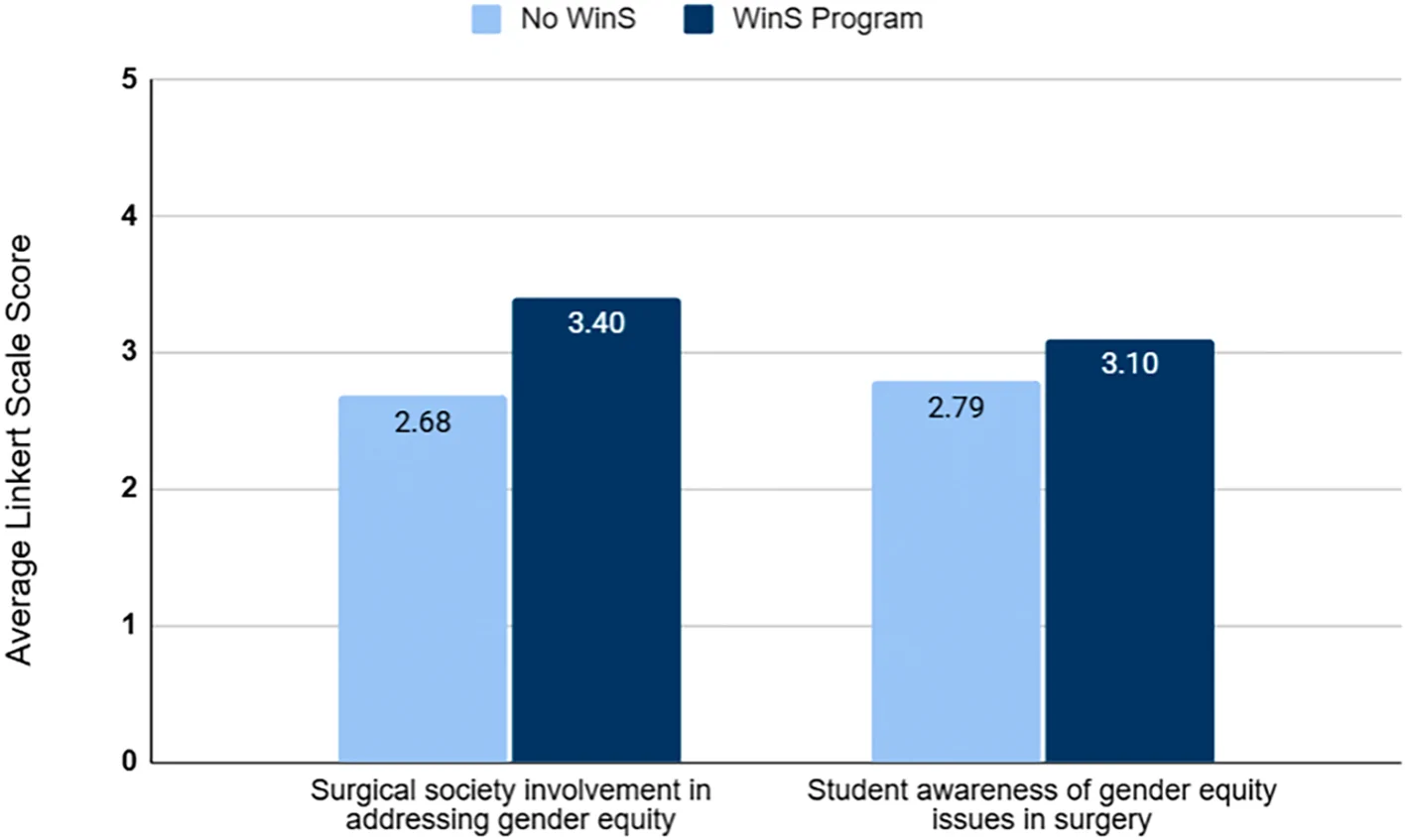
Average Likert scale scores assessing impact of WinS Programs on institutional gender equity.

Institutions with established WinS programs demonstrated significantly stronger engagement with gender equity in surgical education. As outlined in Fig. 1, these universities reported:

• A 26.93% increase in the university surgical society involvement in addressing gender equity (mean Likert rating: 3.40 vs 2.68)
• A 11.28% increase in student awareness of gender equity issues in surgery (mean rating: 3.10 vs. 2.79)

Drawing data from all universities (those with and without WinS), the overall perceived value of WinS initiatives was high, with a mean rating of 4.5 out of 5. Support for cross-institutional collaboration was near-unanimous, with 100% of respondents rating its importance ≥4. When asked whether a WinS-type society was in demand at their medical school, 83.3% responded affirmatively, 8.3% responded negatively, and 8.3% were unsure.

### Key barriers to implementation

**Fig. 2:**
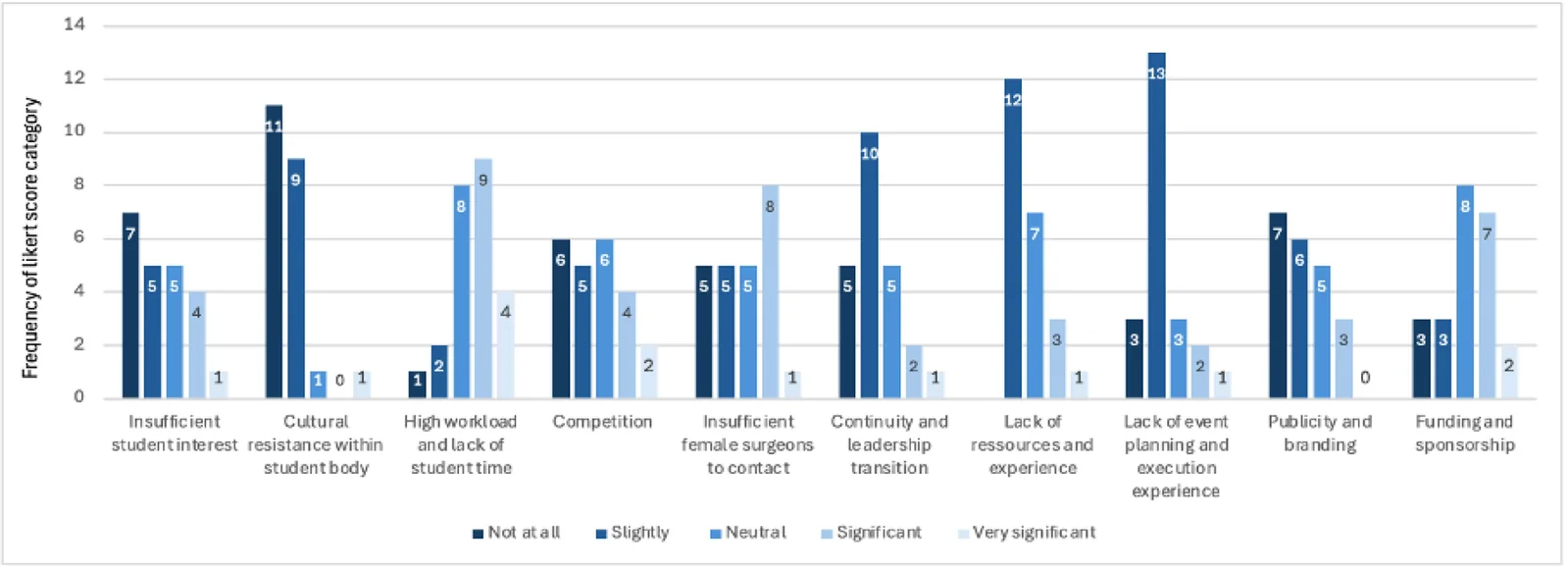
Likert scale scores for barriers experienced by university representatives.

To better understand the specific challenges faced by students in establishing Women in Surgery (WinS) initiatives, we compiled a list of the ten most reported barriers, based on previous student conferences and relevant literature. Participants from each medical school were asked to rate the extent to which each barrier impacted their local context using a 5-point Likert scale (1 = Not significant at all, 5 = Significant barrier). Fig. 2 shows the distribution of responses indicating how many respondents rated each barrier at each level of significance.

When aggregating responses across all 24 participating medical schools, the highest-rated barrier was **high workload and lack of student time** (mean rating: 3.33), followed by **funding and sponsorship barriers** (mean rating: 3.01), and **insufficient access to female surgeons** (mean rating: 2.86). These results summarised in Table 1 provide insight into the perceived limitations most relevant to students and serve as a guide for the prioritisation of support measures aimed at enhancing WinS program development across institutions.

**Table 1:** Average Likert Scale for top 3 out of 10 most common barriers to implementing WinS Program.

Top 3 barriers mentioned	Average Likert scale
1. High workload and lack of student time	3.33
2.Funding and sponsorship barriers	3.01
3.Insufficient female surgeons to contact	2.86

To further contextualise these barriers, we compared responses from schools with active or developing WinS initiatives (*n* = 10) and those without (*n* = 14). As shown in Table 2, both groups identified high workload and time constraints as the most significant challenge. This finding reflects the intensive academic and clinical commitments medical students face, which may hinder their capacity to establish and sustain extracurricular initiatives such as WinS societies.

**Table 2:** Average Likert scale for top 3 out of 10 most common perceived barriers for those with and without existing WinS programs.

No WinS		WinS	
Top 3 barriers mentioned	Average Likert scale	Top 3 barriers mentioned	Average Likert scale
1.High workload and lack of student time	3.14	1.High workload and lack of student time	4.00
2.Insufficient female surgeon to contact	2.71	2. Funding and sponsorship	3.8
3. Funding and sponsorship & 3. Lack of organisational resources and experience	2.57	3. Competition with other societies	3.4

However, divergence emerged in the ranking of secondary barriers. Among schools without a WinS society, **insufficient access to female surgeons** was rated as the second most significant obstacle, indicating that a lack of visible role models and mentors may be a primary deterrent to initiating such programmes. Conversely, students at schools with existing WinS initiatives identified **funding and sponsorship barriers** as their second most significant challenge. This suggests that once a society is operational, the logistical and financial demands of maintaining engagement through events and programming become more pronounced.

These findings underscore the evolving nature of implementation barriers depending on the stage of program development. Institutions in the early stages may require support in fostering visibility and mentorship opportunities, while more established programs benefit from targeted assistance in areas such as funding, event coordination, and long-term sustainability.

### Facilitators identified

**Fig. 3:**
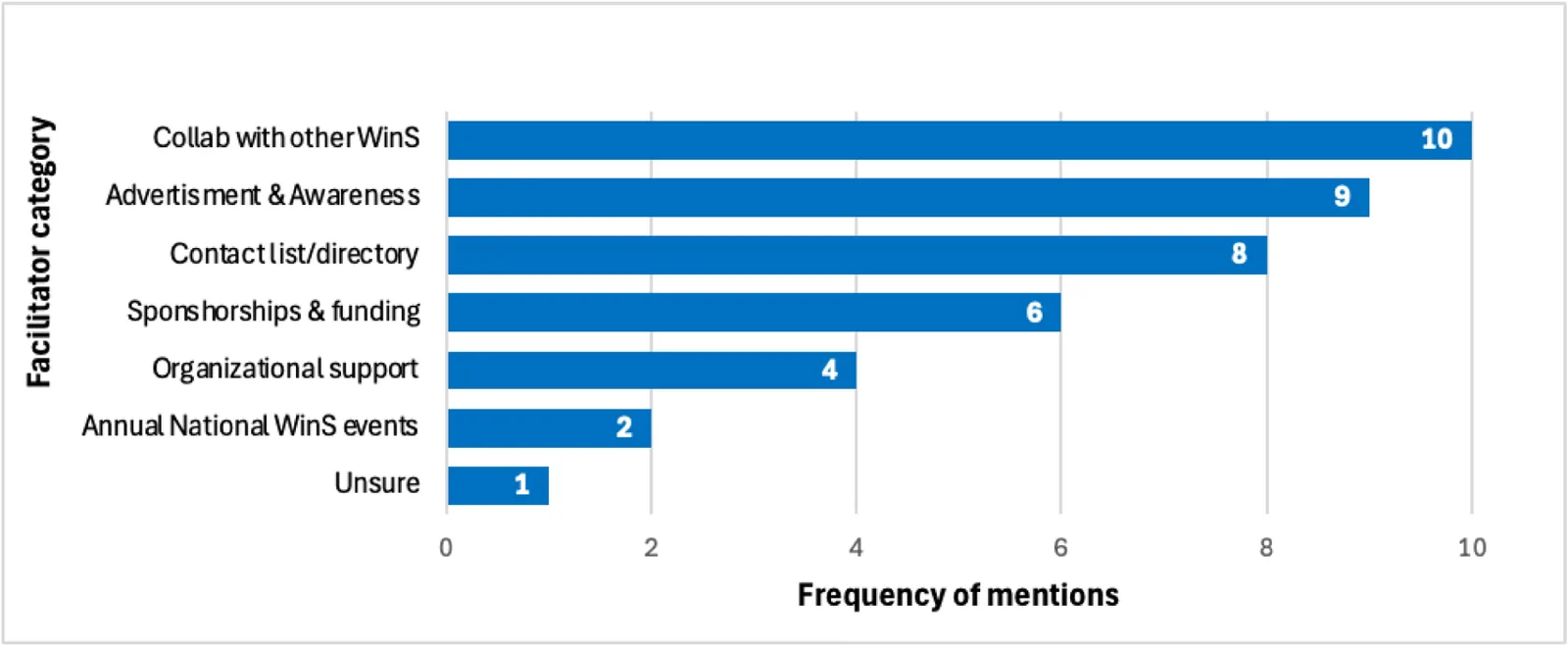
Frequency of facilitator mentions by thematic category.

Qualitative responses highlighted several key facilitators that could support the development and sustainability of WinS programs shown in Fig. 3. The solutions most frequently suggested were:

• Encouraging collaboration across societies and institutions (22.5%)
• Raising awareness of gender inequity in surgical careers and increased advertisement of WinS events (22.5%)
• Providing a reliable directory to facilitate contacting female surgeons for events (20%)

Responses to the open-ended question on support needs were analysed at the university level. The resulting thematic categories of support needs are referred to as facilitators throughout the manuscript and in Fig. 3. Because some institutional representatives were interviewed jointly, each university was treated as a single unit of analysis. Qualitative responses were coded thematically, and a single university could contribute to multiple facilitator categories. Consequently, the total number of coded facilitators mentions exceeded the number of participating universities (*n* = 24). Percentages presented in Fig. 3 therefore reflect the proportion of all coded facilitator mentions represented by each thematic category, rather than the proportion of universities or individual participants.

### Thematic analysis

In addition to quantitative data, open-ended responses were analysed thematically to gain deeper insight into the barriers and facilitators of implementing Women in Surgery (WinS) programmes at UK medical schools. Responses revealed **three dominant themes** which were identified and organised based on the frequency of mention from participant interviews, focusing on both barriers and facilitators:

• (1) Student engagement and cultural perceptions
• (2) Organisational and structural challenges
• (3) Resource and logistical limitations.

Each of these themes encapsulated a spectrum of subthemes, with selected quotes illustrating students' experiences and perspectives. Table 3 shows the different sub themes for the main barriers mentioned in response to open ended questions and Table 4 illustrates the facilitators and types of support that students thought would be most beneficial.

**Table 3:** Thematic analysis for barriers to implementing WinS programs.

Theme	Subthemes	Illustrative Quote(s)
1. Student Engagement and Cultural Perceptions	• Insufficient student interest and low engagement • Cultural resistance or misunderstanding of WinS goals • Misogyny or gender bias • Awareness gap	*“No one has had the initiative to start a WinS. There is a need for people to be proactive and so far, no one has done that”* *“(The biggest barrier is) engagement; gathering enough interest to get committee members as well as other students to be involved with the events”*
2. Organisational and Structural Challenges	• Leadership and continuity issues • Lack of experience and confidence • Administrative and bureaucratic hurdles • Overlap with existing societies- competition	*“A lot of bureaucracy involved in communicating with the student's union, which can be a challenge”* *“The current lead does not have much experience managing finances, will have to learn”*
3. Resource and Logistical Limitations	• Funding and sponsorship issues • Limited access to female surgeons or role models • Venue and scheduling problems • Lack of institutional support	*“Most surgical contacts from the surgical society are male.”* *“There is a lack of female role models, especially in areas such as orthopaedic surgery”*

**Table 4:** Thematic Analysis for facilitators to implementing WinS programs.

Theme	Subthemes	Illustrative Quote(s)
1. Student Engagement and Cultural Perceptions	• Increase awareness through publicity and visibility • Dedicated publicity roles in committees • Advice on strategies to increase events engagement • Broader audience profiles and inclusivity • Gradual introduction of small events • Confidence building • Promoting importance • Safe space messaging • Networks and collaboration	*“Raising awareness. Hearing female surgeons talk about what drew them to that career as well as how they've managed to balance it with the rest of their life. It's good to have introductory talks about pros and cons of a career in surgery, in addition to portfolio-oriented talks.”*
2. Organisational and Structural Challenges	• Guide on society setup & admin process • Detailed committee handovers to ensure continuity • Help from student union for coordination • Collaboration of events with other societies to avoid competition • Guidance from umbrella organisations (e.g. WinSSA)	*“Communicating with WinSSA directly to get advice on setting up a society”*
3. Resource and Logistical Limitations	• Directory for access to surgeon's contacts • Funding and sponsorship contacts – makes access easier • Support networks to ask for advice • Shared resources across universities • Increase number of students on committee	“Getting access to surgeons and contacts would make things easier”

## Discussion

### Student engagement and cultural perceptions

Many participants cited insufficient motivation among students to initiate or sustain WinS societies. A recurring issue was the lack of proactive leadership: *“No one has had the initiative to start a WinS. There is a need for people to be proactive and so far no one has done that.”* This lack of engagement extended beyond leadership, with organisers facing difficulty in recruiting committee members or attracting participants to events. Similar challenges have been reported in other areas of medical education. Mahmood et al. (2025) [5] reported that undergraduate medical students faced multiple barriers engaging in research-related activities including limited motivation, competing priorities, and difficulties sustaining participation. These findings suggest that challenges in student engagement may extend beyond surgical initiatives and represent a broader issue within extracurricular and professional development activities in medical education.

Participants linked low engagement to perceptions of surgery as a “*competitive and male-dominated”* field, leading to reduced interest among female students, particularly those in pre-clinical years. *“Not enough people are interested... A lot of people don't consider surgery because it's too competitive and male-dominated.”*

Deeper cultural barriers also emerged, including underlying gender biases and perceptions that WinS initiatives are unnecessary, especially among male students or those unaware of the challenges women face in surgery. Some schools reported a history of resistance to gender-focused efforts: *“[We] had issues with gender equality in the past... there will always be people asking if it's necessary to have a WinS.”* These sentiments on the myth of meritocracy are echoed by Vela et al. (2023) [6], who identified a prevailing culture in surgery that dismisses equity initiatives as unnecessary, based on the flawed belief that the field is a pure meritocracy. These attitudes, combined with imposter syndrome and a lack of visible role models, hinder efforts to create inclusive, engaging spaces. This highlights the need to raise awareness while fostering inclusivity, correcting misconceptions, and encouraging peer support.

Workforce-level data further contextualise these cultural barriers. Begeny et al. (2023) [7] documented alarming rates of sexual misconduct among surgical professionals, with women disproportionately affected. This reflects the deeply entrenched gender dynamics students may anticipate entering and reinforces the case for early intervention. Initiatives like WinS, embedded at medical school level, are well-positioned to begin shifting cultural norms before students enter the workforce.

Suggested facilitators in this area included skill-based workshops like suturing or simulation sessions to draw wider interest. Events with broader themes, such as talks on imposter syndrome, mentorship, or Q&As with approachable surgeons, can engage students of all genders and levels of commitment. Creating an open, welcoming environment with safe-space messaging builds inclusion, while increased visibility through social media, newsletters, and hybrid events makes participation easier for busy students. Collaboration with national bodies like WinSSA (Women in Surgery Student Association) and other universities can also enhance resources and impact.

### Organisational and structural challenges

This category highlighted a lack of institutional recognition and bureaucratic hurdles, especially in new or smaller medical schools: *“It's difficult for a new society to get students to take on lead roles because the society isn't established yet... all the work needs to be done from scratch.”* Many felt isolated in their efforts to set up events due to a lack of clear guidance or support from medical schools.

Administrative hurdles were exacerbated by student leaders' inexperience with finances and event organisation, and by inconsistent committee handovers that weakened continuity. Participants consistently described a pattern of ‘annual reinvention’, whereby insufficient handover processes between outgoing and incoming committee members resulted in repeated loss of institutional knowledge, contacts, and momentum.

Students also cited competition with pre-existing surgical or women-in-healthcare societies as a deterrent: *“There is already a surgical society, which is well known... people hesitate to join multiple societies due to cost and overlap.”*

These organisational barriers undermine momentum and make it harder to build a sustained, well-supported WinS presence on campus.

Respondents suggested clearer guidelines from student unions and stronger support from medical schools to ease the formation of WinS societies. Early recruitment of first and second-year students, along with defined committee roles, were seen as key to continuity and workload distribution. Sharing best practices through national events, forums, or WinSSA resource packs, along with thorough handovers and mentorship, allows new leaders to learn from more experienced peers. Some suggested initially hosting WinS events within established surgical societies to leverage existing resources and build momentum.

Finally, near-unanimous support (100% of respondents rating ≥ 4) for cross-institutional collaboration and a national framework is a powerful finding. It strongly aligns with calls from organisations like the Royal College of Surgeons for a coordinated, national approach to tackling gender inequity.

### Resource and logistical limitations

A key challenge in this category was the limited access to female surgical mentors, especially in traditionally male-dominated specialties like orthopaedics: *“Most surgical contacts from the surgical society are male.”* This barrier was particularly pronounced in universities without established WinS programmes, where a lack of visible role models compounded students' uncertainty about how to engage.

The shortages of accessible female role models mirror findings by Hill et al. (2013) [8], who identified the scarcity of visible female surgeons as a significant deterrent for students considering a surgical career. This “you can't be what you can't see” phenomenon is a well-established barrier to diversifying surgical fields [9].

Our quantitative finding that institutions with WinS programs reported significantly higher engagement in gender equity (mean rating: 3.40 vs. 2.68) supports the growing evidence that targeted interventions can effectively shift institutional culture. This is supported by Kerdegari et al. (2024) [10], who demonstrated in a prospective cohort study that surgical mentorship significantly improved medical students' surgical experience and attitudes towards pursuing surgery as a career. This suggests that targeted mentorship initiatives like WinS may yield similar benefits for female students specifically.

Other limitations included difficulty securing funding and sponsorship for events, finding appropriate venues, scheduling events around a demanding academic calendar, and insufficient institutional support. Financial constraints are particularly present for newer societies: *“As a new society, we don't get SU funding in the first year... most funding comes from membership money.”* Even when student interest existed, these resource-related hurdles often prevented progress.

Facilitators included leveraging WinSSA's national network, creating a central contact directory, strengthening student-mentor connections, and engaging supportive clinicians early. Funding could come from medical company sponsorships, hospital diversity funds, event-specific student union grants, or collaboration with established societies like Women in Medicine or the Surgical Society.

### Overview and recommendations

The three themes explored above often interact and converge. Low student engagement can stem from cultural perceptions and insufficient institutional or financial support, while organisational barriers like poor committee handover exacerbate resource challenges by limiting knowledge transfer and network access. These findings highlight the need for a comprehensive support system that addresses awareness, logistics, and infrastructure in a coordinated manner.

This study also highlights a nuanced evolution of barriers. The divergence in perceived challenges where schools with WinS ranked funding as secondary, while those without cited access to surgeons, suggesting difficulties change with a program's lifecycle. This adds a new layer to the literature, indicating that support frameworks must be dynamic and stage-specific, offering mentorship access for nascent groups and financial/structural support for established ones.

Future research should evaluate the long-term impact and sustainability of WinS programs in different medical school. It would be interesting to conduct longitudinal follow-ups to assess changes in student engagement, perceptions, and career outcomes over months or years. Additionally, identifying transferable lessons from past WinS initiatives can help to develop scalable, context-sensitive strategies.

### Strengths

This study has several key strengths. Its robust cross-sectional design incorporates both qualitative and quantitative methods, combining open-ended interview questions with Likert-scale ratings which captures nuanced insights alongside measurable trends. With a diverse sample of participants from across UK medical schools, including current and former society leaders, the study reflects a range of perspectives. Data analysis was conducted independently by multiple researchers, with discrepancies resolved collaboratively, enhancing the reliability and credibility of identified themes. Importantly, the study explores topics that are both highly relevant and under-researched, providing valuable evidence on the barriers and facilitators of WinS initiatives in a context that has received limited prior attention.

### Limitations

This study has limitations. The sample, while substantial, included 24 of 45 UK medical schools; the perspectives of non-participating schools may differ, limiting generalisability. Our analysis of Likert scale responses using the Mann–Whitney *U* test did not show statistically significant differences. This lack of statistical significance may reflect a true absence of difference, but it could also be due to factors such as sample size, variability within responses, or insufficient power to detect smaller effects. Furthermore, the study relies on self-reported data, which introduces the possibility of bias, including recall bias and social desirability bias. Subjective factors such as individual experiences, stage of training (pre-clinical vs. clinical years), and exposure to surgical disciplines may have influenced responses.

## Conclusion

This study identified key barriers to implementing WinS programs, including resource limitations and logistical challenges, while emphasising the positive impact of mentorship and collaboration. With only 42% of schools offering WinS, strategic interventions like a national resource directory and funding are crucial to supporting female medical students in surgery.

Addressing gender inequity in surgery is an urgent and actionable priority. Medical schools, policymakers, and surgical societies must work collaboratively to fund, implement, and sustain WinS initiatives - empowering the next generation of female surgeons and shaping a more representative surgical landscape.

To achieve lasting change, a grassroots approach is essential: we must begin targeting students at the earliest stages of their medical education, encouraging more women to consider surgical careers and proactively dismantling perceived barriers before they become limiting beliefs.

## CRediT authorship contribution statement

**Samruddhi Tagalpallewar:** Writing – review & editing, Writing – original draft, Visualization, Supervision, Resources, Project administration, Methodology, Investigation, Formal analysis, Data curation, Conceptualization. **Lara Melloul:** Writing – review & editing, Writing – original draft, Visualization, Supervision, Resources, Project administration, Investigation, Formal analysis, Data curation. **Oliwia Bogucka:** Data curation. **Lucy Bowden:** Data curation. **Arul Nilaa Arul Jothi:** Data curation. **Ira Makhijani:** Data curation. **Ranoo Rebaz:** Data curation. **Polina Zabelina:** Formal analysis.

## Financial disclosure statement

This research did not receive any specific grant from funding agencies in the public, commercial, or not-for-profit sectors.

## Declaration of competing interest

The authors declare that they have no known competing financial interests or personal relationships that could have appeared to influence the work reported in this paper.
